# The Cytoscape app article collection

**DOI:** 10.12688/f1000research.4642.1

**Published:** 2014-07-01

**Authors:** Alexander R Pico, Gary D Bader, Barry Demchak, Oriol Guitart Pla, Timothy Hull, William Longabaugh, Christian Lopes, Samad Lotia, Piet Molenaar, Jason Montojo, John H Morris, Keiichiro Ono, Benno Schwikowski, David Welker, Trey Ideker

**Affiliations:** 1 Gladstone Institutes, San Francisco, CA 95158, USA; 2 The Donnelly Centre, University of Toronto, Toronto, ON M5S 3E1, Canada; 3 University of California, San Diego, La Jolla, CA 92093, USA; 4 Institut Pasteur, Paris, 75015, France; 5 Institute for Systems Biology, Seattle, WA 98109, USA; 6 Amsterdam Medical Centre, Amsterdam, 1105 AZ, Netherlands; 7 University of California San Francisco, San Francisco, CA 94143, USA

## Abstract

As a network visualization and analysis platform, Cytoscape relies on apps to provide domain-specific features and functions. There are many resources available to support Cytoscape app development and distribution, including the Cytoscape App Store and an online “cookbook” for app developers. This article collection is another resource to help researchers find out more about relevant Cytoscape apps and to provide app developers with useful implementation tips. The collection will grow over time as new Cytoscape apps are developed and published.

## Editorial

Cytoscape is an open source software platform for network visualization, data integration and network analysis ^1– 3^. Cytoscape is most commonly applied to molecular networks and pathways in biology, though it is also used to interrogate the entire range from atomic and residue interactions in protein structures to chromosomal, cellular, organismal and social networks, as well as non-biological applications. Its success as a research tool largely comes from its flexibility. By maintaining an agnostic approach toward specific data types, file formats and network semantics, Cytoscape enables researchers to target any particular area of study they choose. This is accomplished through the development and use of Cytoscape apps.

Cytoscape apps are software applications written in Java that customize and extend the functionality of Cytoscape. There are apps that generate networks in Cytoscape based on external sources of interactions and biological data; there are apps that perform integrated analysis with multiple data types, cluster analysis and graph analysis based on network topology; there are also apps that produce automatic and custom data visualizations ^4^. In total, over two hundred apps contributed by developers around the world are freely available at the Cytoscape App Store ^5^ ( http://apps.cytoscape.org). Researchers can browse and search for apps by name, keyword or category, compare download and ranking statistics, find links to tutorials and manuals, and even install apps with a single click from within the browser. The site benefits app developers as well by not only distributing their software, but also providing page editing tools, download statistics over time and geography, and links to open source code repositories for other apps. Additional resources for the growing community of app developers can be found at http://wiki.cytoscape.org/Cytoscape_3/AppDeveloper, including documentation on the redesigned architecture and API of Cytoscape 3, app developer tutorials and a code snippet “cookbook”.

This collection of Cytoscape app articles at *F1000Research* is intended to serve as a resource to both researchers and app developers. The author guidelines were customized for this collection to encourage *relevant* use cases and *useful* implementation details in a short article format. Researchers will find apps that connect to KEGG, Reactome and WikiPathways, perform identifier mapping, interface with R, GenomeSpace and GeneMANIA, perform network alignment and topological analysis, add pie charts to nodes, and export networks to the Web. App developers will find descriptions of Cytoscape API usage, including *TaskIterator*, *CyNetworkReader, VisualMappingManager* and *CyCustomGraphics*, as well as command interfaces exposed by other apps and links to their code. Every Cytoscape app published in this collection is free and open source.

This article collection is a tailored publication hub for new and updated Cytoscape apps. Track this collection for new articles as developers continue to produce and publish new Cytoscape apps.

## References

[1] Shannon P Markiel A Ozier O: Cytoscape: a software environment for integrated models of biomolecular interaction networks. *Genome Res.* 2003;13(11):2498–2504. 10.1101/gr.1239303 14597658 PMC403769

[2] Cline MS Smoot M Cerami E: Integration of biological networks and gene expression data using Cytoscape. *Nat Protoc.* 2007;2(10):2366–2382. 10.1038/nprot.2007.324 17947979 PMC3685583

[3] Smoot ME Ono K Ruscheinski J: Cytoscape 2.8: new features for data integration and network visualization. *Bioinformatics.* 2011;27(3):431–432. 10.1093/bioinformatics/btq675 21149340 PMC3031041

[4] Saito R Smoot ME Ono K: A travel guide to Cytoscape plugins. *Nat Methods.* 2012;9(11):1069–1076. 10.1038/nmeth.2212 23132118 PMC3649846

[5] Lotia S Montojo J Dong Y: Cytoscape app store. *Bioinformatics.* 2013;29(10):1350–1351. 10.1093/bioinformatics/btt138 23595664 PMC3654709

